# The anti-tumor efficacy of CDK4/6 inhibition is enhanced by the combination with PI3K/AKT/mTOR inhibitors through impairment of glucose metabolism in TNBC cells

**DOI:** 10.1186/s13046-018-0741-3

**Published:** 2018-03-27

**Authors:** Daniele Cretella, Andrea Ravelli, Claudia Fumarola, Silvia La Monica, Graziana Digiacomo, Andrea Cavazzoni, Roberta Alfieri, Alessandra Biondi, Daniele Generali, Mara Bonelli, Pier Giorgio Petronini

**Affiliations:** 1 Department of Medicine and Surgery, University of Parma, Parma, Italy; 2 Department of Medical, Surgery and Health Sciences, University of Trieste, Trieste, Italy; 3 U.O. Multidisciplinare di Patologia Mammaria, U.S Terapia Molecolare e Farmacogenomica, ASST Cremona, Cremona, Italy

**Keywords:** Palbociclib, Triple-negative breast cancer, CDK4/6 inhibition, PI3K/mTOR inhibitors, Glucose metabolism

## Abstract

**Background:**

Cell cycle regulators have gain attention as potential targets for anticancer therapy. Palbociclib is a selective inhibitor of the cyclin-dependent kinases 4 and 6 (CDK4/6), which coordinate the G1-S transition. Palbociclib is currently approved for the treatment of hormone receptor positive, HER2-negative advanced breast cancer (BC) in association with letrozole or fulvestrant. In contrast, its efficacy in triple negative BC (TNBC), either alone or in combined therapies, has not been fully investigated to date.

**Methods:**

Here we evaluated the potential of combining palbociclib with PI3K/mTOR inhibitors in Rb-proficient TNBC cells comparing different schedules of treatment: simultaneous, sequential, or sequential combined treatment (pre-incubation with palbociclib followed by exposure to both palbociclib and PI3K/mTOR inhibitors). We assessed the effects on cell proliferation, cell death, and cell cycle distribution, and looked at the impact of such treatments on glucose metabolism.

**Results:**

Palbociclib exerted cytostatic effects in Rb-positive TNBC cells, inducing a reversible blockade in G0/G1 cell cycle phase associated with down-regulation of CDK6, Rb, and c-myc expression and/or activity. Palbociclib treatment induced AKT signaling, providing a rationale for its combination with PI3K/mTOR inhibitors. The simultaneous or sequential treatment resulted in an additive inhibition of cell proliferation. On the other hand, the sequential combined treatment in which palbociclib was maintained also during exposure to PI3K/mTOR inhibitors gave rise to synergistic anti-proliferative and pro-apoptotic effects, by inhibiting both CDK4/6/Rb/myc and PI3K/mTOR signaling. Interestingly, the inhibition of the Rb/E2F/myc axis mediated by palbociclib resulted in a significant down-regulation of glucose metabolism; most importantly, these inhibitory effects were enhanced by the combination of palbociclib with BYL719 (specific inhibitor of the p110α PI3K-subunit), which promoted a stronger inhibition of GLUT-1 glucose transporter expression, glucose uptake and consumption in comparison with individual treatments, under both normoxic and hypoxic conditions.

**Conclusions:**

Combination of palbociclib with PI3K/mTOR inhibitors may represent a promising therapeutic option for the treatment of Rb-proficient TNBC, with the sequential combined schedule showing a superior efficacy over the other schedules. In addition our results demonstrate that the impairment of glucose metabolism may contribute to the anti-tumor activity of such drug combinations.

## Background

In spite of the multitude of pharmacologic approaches which have become clinically available during the last decades and novel screening improvements, breast cancer (BC) remains the second leading cause of cancer-related death among women [1]. BC subtypes are based on the expression of hormone receptors, i.e. estrogen receptor (ER) and/or progesterone receptor (PR) (∼75% of cases), and overexpression/amplification of the human epidermal growth factor receptor 2 (HER2) (∼20% of cases, half of which are also positive for hormone receptors). Tumors lacking the expression of such receptors are commonly referred to as Triple-negative BCs (TNBCs) (∼5%–10%) [2]. In addition, the development of gene expression profiling using high-throughput analysis has provided a molecular classification of BC into luminal A, luminal B, HER2-enriched, basal-like, claudin-low, and normal-like subtypes [3]. TNBCs are mostly basal-like and are associated with high aggressiveness and poor prognosis. Due to the lack of druggable targets, treatment of TNBC is based on chemotherapy and the identification of new targets is a high clinical priority.

p16^INK4^ is a cyclin-dependent kinase inhibitor (CDKI), that blocks the binding site of cyclin D1 on CDK4/6. Loss of functional p16^INK4^ gives rise to deregulated CDK4/6 activity, leading to persistent retinoblastoma protein (Rb) phosphorylation and increasing cell proliferation [4]. The loss of p16^INK4^ has been reported to occur with higher frequency in TNBC in comparison with other BC histotypes and has been correlated with the poor prognosis of TNBC [5]. In addition, the lack of p16^INK4^ expression has been associated with the acquisition of cancer stem cell-like properties and with a reduced response of TNBC to paclitaxel treatment [6]. Also the inactivation of Rb, due to both mutation or homozygous loss of the gene, may be observed in all BC subtypes, with a higher frequency in TNBC (7–20%) [7, 8].

Palbociclib, an orally-available inhibitor of CDK4 and CDK6, represents the most widely studied compound among cell cycle inhibitors. Palbociclib is a cytostatic drug, which efficiently blocks cell cycle progression from G1 to S phase by preventing the CDK4/6-cyclin D1-mediated phosphorylation of Rb and the subsequent release of the transcription factor E2F [9].

Palbociclib granted accelerated approval in 2015 for the treatment of ER-positive, HER2-negative advanced BC in association with letrozole [10], and in combination with fulvestrant in patients with ER-positive/HER2-negative advanced BC with disease progression following endocrine therapy [11]. Intriguingly, some early preclinical evidences have been documenting a possible efficacy of palbociclib in other BC molecular subvariants, including TNBC cell lines [12]. Based on the aforementioned considerations, TNBC with a Rb-positive, p16^INK4^-negative profile might represent the subpopulation of TNBC suitable for treatment with palbociclib.

In addition to a direct effect of palbociclib on sensitive malignant cells, the mechanism of action of the drug also suggests a possible role for combinatory schedules of treatment. In particular, the recently described mechanism of palbocicilib-mediated activation of Protein Kinase B (AKT) signaling [13] provides a strong rationale for the combination with inhibitors of the phosphoinositide 3-kinase/AKT/mammalian target of rapamycin (PI3K/AKT/mTOR) pathway. This pathway plays a critical role in the control of cell growth, proliferation, migration, and metabolism, and, being frequently deregulated in BC cells, PI3K/mTOR inhibitors are under evaluation in numerous clinical trials [14].

These preliminary observations represent the bases of the present work, designed to evaluate the activity of palbociclib on a panel of TNBC cell lines with regards to the possible utilization of the drug also in association with PI3K/mTOR inhibitors. Our findings demonstrated that the combination of palbociclib with PI3K/mTOR inhibitors enhances the growth inhibitory effects of the single agents, and impairs tumor cell metabolism, suggesting new therapeutic strategies to challenge the aggressive behavior of TNBC.

## Methods

### Cell culture

Human BC cell lines MDA-MB-231, MDA-MB-468, HCC-38 (all triple negative) and MCF-7 (ERα-positive) were cultured in RPMI supplemented with 2 mM glutamine, 10% fetal bovine serum (FBS), and 100 U/ml penicillin/100 μg/ml streptomycin.

Cells were purchased from the American Type Culture Collection (Manassas, VA), which authenticates the phenotypes of these cell lines on a regular basis (http://www.lgcstandards-atcc.org). Hypoxic conditions were established by placing the cells in a tissue culture incubator with controlled O_2_ levels (Binder GmbH, Tuttlingen, Germany).

### Drug treatment

Palbociclib (PD-0332991) was provided by Pfizer (New York City, NY); NVP-BEZ235, NVP-BYL719, and NVP-BKM120 (hereafter, referred to as BEZ235, BYL719, and BKM120) were provided by Novartis Institutes for BioMedical Research (Cambridge, MA). Drugs were prepared in DMSO, and DMSO concentration never exceeded 0.1% (*v*/v); equal amounts of the solvent were added to control cells.

### Analysis of cell proliferation, cell death and cell cycle

Cell proliferation was evaluated by counting the cells in a Bürker hemocytometer with trypan blue exclusion method and by Crystal Violet (CV) staining as previously described [15]. The nature of the interaction between palbociclib and PI3K inhibitors was calculated using the Bliss additivism model [16]. A theoretical dose-response curve was calculated for combined inhibition using the equation of Bliss = EA + EB-EA*EB, where EA and EB are the percent of inhibition versus control obtained by BYL719, BEZ235 or BKM120 (A) and palbociclib (B) alone and the E Bliss is the percent of inhibition that would be expected if the combination was exactly additive. If the combination effect is higher than the expected Bliss equation value, the interaction is synergistic, while if the effect is lower, the interaction is antagonistic. Otherwise, the effect is additive and there is no interaction between the drugs. Cell death was analyzed by fluorescence microscopy after staining with Hoechst 3342 and Propidium Iodide (PI) [17]. Distribution of the cells in the cell cycle was determined as previously described [18]. Briefly, 5 × 10^5^ cells were incubated overnight at 4 °C in 1 ml of hypotonic fluorochrome solution. Analysis was performed with Coulter EPICS XL-MCL cytometer (Coulter Co., Miami, FL, USA). Cell-cycle-phase distributions were analyzed by MultiCycle DNA Content and FCS Express Software (De Novo Software, Glendale, CA 91203).

### Western blotting

Analyses of western blotting were performed as previously described [19].

Antibodies against p-Rb^Ser780^ (#9307), Rb (#9309), cyclin D1 (#2926), CDK6 (#3136), c-myc (#9402), p-AKT^Ser473^ (#9271), AKT (#9272), p-mTOR^Ser2448^ (#2971), mTOR (#2972), p-ERK1/2^Thr202/Tyr204^ (#4370), ERK1/2 (#4695), were from CST (Danvers, MA); anti-p-CDK6^Tyr24^ (sc-293,097) was from Santa Cruz Biotechnology, Incorporated (Dallas, TX). Anti CDKN2A/p16^INK4a^ (ab81278) and anti-GLUT-1 (ab40084) were from Abcam (Cambridge, UK). Antibody against HIF-1α (#610959) was from BD Biosciences (Franklin Lakes, NJ). Anti-β-actin (#3598) was from BioVision (Milpitas, CA). All the antibodies were used at the recommended dilution of 1:1000. Horseradish peroxidase-conjugated secondary antibodies (1:10,000) and chemiluminescence system were from Millipore (Millipore, MA). Reagents for electrophoresis and blotting analysis were from BIO-RAD Laboratories (Hercules, CA).

### Quantitative real-time PCR

Total RNA was isolated by RNeasy Mini Kit (Qiagen, Venlo, Netherlands) and the quantitative real-time polymerase chain reaction (PCR) was performed using the StepOne system instrument (Applied Biosystems) as previously described [20]. Briefly, samples were amplified using the following thermal profile: 95 °C for 20 s and 40 cycles of denaturation at 95 °C for 3 s followed by annealing and extension at 60 °C for 30 s. The primer to specifically amplify GLUT-1 (QT00068957) was obtained from Qiagen. HPRT1 (QT00059066) and PGK1 (QT00013776) were used as housekeeping genes and were purchased from Qiagen. The fold change was calculated by the ΔΔCT method.

### Glucose uptake and consumption

Glucose uptake was measured as described previously [21]. Briefly, cells were rinsed with Kreb’s Ringer HEPES buffer (KRH) and incubated in KRH containing 1 μCi/ml Deoxy-D-glucose-2-[1,2-3H(N)] (2DG, PerkinElmer, Waltham, MA) at 37 °C for 5 min. Then, the cells were quickly rinsed three times in fresh cold Earle’s solution containing 0.1 mM phloretin (Sigma-Aldrich). Ice-cold trichloroacetic acid (TCA, 5%) was added and radioactivity in the acid extracts was measured by liquid-scintillation. Cell proteins, precipitated by TCA, were dissolved in 0.5 N NaOH and their concentration determined by a dye-fixation method (Bio-Rad, Hercules,CA). Glucose uptake was calculated as pmol of 2DG/mg protein/5 min and expressed as percent vs control condition. Glucose levels in the media were determined using a Glucose (HK) Assay Kit (product code GAHK-20) (Sigma-Aldrich, St. Louis, MI), according to the manufacturer’s instruction. Glucose consumption was calculated subtracting the glucose amount in the spent media to glucose in cell-free media. Data were calculated as mg glucose/mg protein and expressed as percent vs control.

### Statistical analysis

Statistical analyses were carried out using GraphPad Prism 6.00 software. Statistical significance of differences among data was estimated by two-tailed Student’s t test. Comparison among groups was made using analysis of variance (one-way ANOVA, repeated measures) followed by Tukey’s post-test.

## Results

### Effects of palbociclib on cell proliferation and cell cycle distribution in TNBC cells

**Fig. 1:**
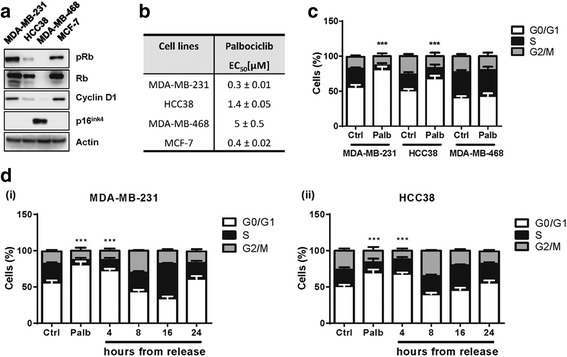
Palbociclib inhibits cell proliferation through G0/G1 cell cycle arrest. **a** The expression of cell cycle-related proteins was analyzed by Western blotting in TNBC and ERα + cells 24 h after seeding. **b** TNBC and ERα + cell lines were treated with increasing concentrations of palbociclib (0.01-10 μM); after 72 h cell proliferation was evaluated by CV staining. The EC_50_ values are shown. **c** TNBC cells were treated in absence (ctrl) or presence of 0.5 μM palbociclib. After 24 h the distribution of cells in cell cycle phases was determined by flow cytometry. **d** MDA-MB-231(i) and HCC38 (ii) cells were treated with palbociclib 0.5 μM and 1 μM, respectively. After 24 h palbociclib was removed and cell cycle phase distributions were evaluated by flow cytometry at the indicated time points. ***p < 0.001 vs corresponding G0/G1 ctrl. Results in **a** are representative of three independent experiments. Data in **b**-**d** are means ±SD of three independent experiments (*n* = 5)

We firstly evaluated the effect of palbociclib on cell proliferation in a panel of TNBC cell lines (MDA-MB-231, MDA-MB-468, HCC38) in comparison with a ER+ luminal-A BC cell line (MCF-7), that reflects the cancer histotype for which the drug is currently clinically used. Cell sensitivity to palbociclib can be predicted by the presence of detectable levels of p-Rb and cyclin D1, and by a contemporary reduced expression of the cell cycle inhibitor p16^INK4^ [22]. As shown in Fig. 1a, MDA-MB-231, HCC38, and MCF-7 cell lines expressed both p-Rb as well as cyclin D1, whereas p16^INK4^ was not detectable; accordingly, palbociclib inhibited cell proliferation in these cell models with EC_50_ values ranging from 0.3 to 1.4 μM (Fig. 1b). In contrast, MDA-MB-468 cells, characterized by the loss of Rb and low expression of cyclin D1, while showing p16^INK4^ expression, were less sensitive to palbociclib (Fig. 1a,b). In addition, palbociclib elicited a robust cell cycle blockade in G0/G1 phase only on sensitive cell models (80 and 70% of cells in G0/G1 phase for MDA-MB-231 and HCC38, respectively), whereas MDA-MB-468 cells did not show any variation in the distribution of cells within cell cycle phases (Fig. 1c).

**Fig. 2:**
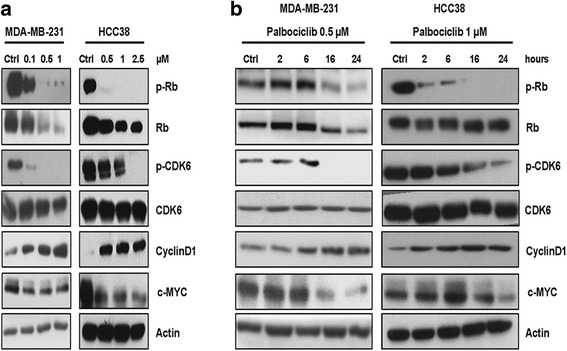
Palbociclib modulates the activation/expression of cell cycle-related proteins in a dose- and time-dependent manner. MDA-MB-231 and HCC38 cells were treated with increasing concentrations of palbociclib for 24 h (**a**) or with a fixed drug concentration for different periods of time (**b**). The expression of the indicated proteins was analyzed by Western blotting. Results are representative of three independent experiments

Then, to investigate the reversibility of palbociclib on cell cycle, we treated MDA-MB-231 and HCC38 cells with palbociclib for 24 h, after which the compound was removed and cell distribution within cell cycle phases was analyzed at increasing time intervals. After drug removal, the initial blockade in G0/G1 phase was progressively lost and after 24 h the normal distribution of cells in each cell cycle phase was restored in both cell lines, thus confirming that the growth-inhibitory effect of palbociclib is reversible (Fig. 1d). We then evaluated the effect of palbociclib on the expression of cell cycle-related proteins and observed that the treatment induced a decrease of p-Rb, Rb and p-CDK6 levels and a concomitant increase of cyclin D1 levels in a dose- and time-dependent manner (Fig. 2a,b, respectively). Hypophosphorylation of Rb protein, due to CDK4/6 inhibition, is known to result in its activation; active Rb maintains E2F in a repressed state, with consequent inhibition of downstream gene transcription [8]. Indeed, palbociclib treatment of MDA-MB-231 and HCC38 cells down-regulated the expression of myc, which contains E2F-binding sites in its regulatory region (Fig. 2a,b).

### Effects of palbociclib in combination with PI3K/mTOR inhibitors

**Fig. 3:**
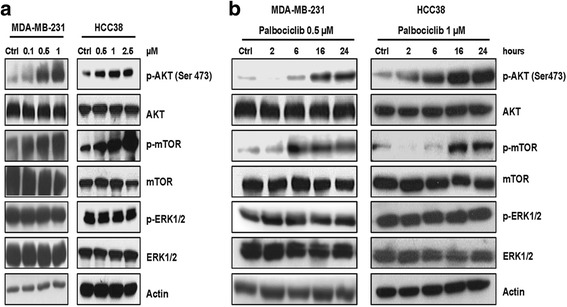
Palbociclib up-regulates the PI3K/AKT/mTOR pathway in TNBC cells. MDA-MB-231 and HCC38 cells were treated with increasing concentrations of palbociclib for 24 h (**a**) or with a fixed drug concentration for different periods of time (**b**). Then, the expression of the indicated proteins was analyzed by Western blotting. Results are representative of three independent experiments

Since it has been recently reported that palbociclib treatment increases the activation of AKT/mTOR signaling [13, 23], we evaluated the activation status of this pathway in MDA-MB-231 and HCC38 cells after dose- and time-dependent exposure to palbociclib (Fig. 3a,b). Palbociclib induced a dose-dependent up-regulation of the phosphorylation levels of mTOR and AKT proteins. On the contrary, no regulatory effects were observed for ERK-1/2 pathway. The activation of both AKT and mTOR increased also over time up to 24 h.

**Fig. 4:**
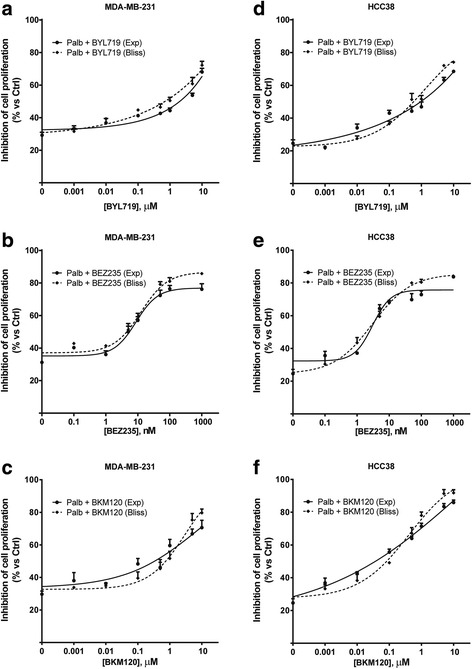
A simultaneous treatment with palbociclib and PI3K/AKT/mTOR inhibitors induces additive effects. MDA-MB-231 (**a**-**c**) and HCC38 cells (**d**-**f**) were treated with 0.5 μM palbociclib alone or in combination with increasing concentrations of BYL719, BEZ235 or BKM120. After 72 h cell proliferation was assessed by CV assay. The effect of the drug combinations was evaluated using the Bliss interaction model. Data are mean values ±SD of three independent experiments (*n* = 5)

**Fig. 5:**
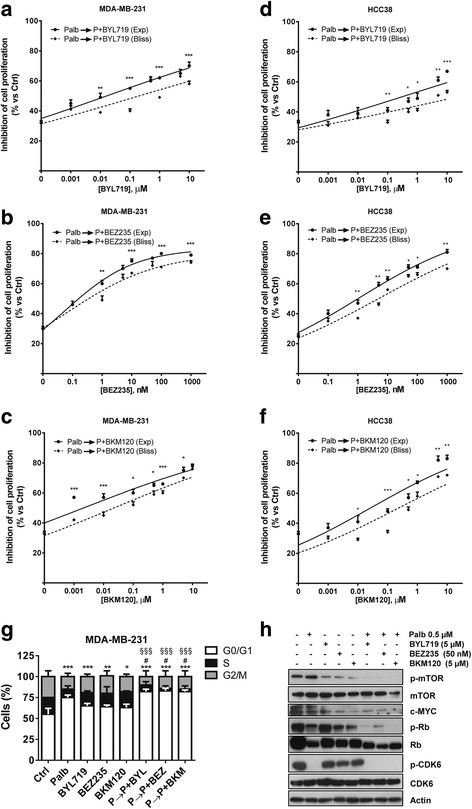
A sequential exposure to palbociclib and PI3K/AKT/mTOR inhibitors, with palbocilcib maintained during all the treatment, induces synergistic effects. MDA-MB-231 (**a**-**c**) and HCC38 cells (**d**-**f**) were pre-incubated with 0.5 μM palbociclib for 24 h. Then, the cells were treated with increasing concentrations of BYL719, BEZ235 or BKM120 alone or in combination with palbociclib. After 48 h cell proliferation was assessed by CV assay. The effect of the drug combinations was evaluated using the Bliss interaction model. Data are mean values ±SD of three independent experiments (n = 5). **g** MDA-MB-231 cells were treated with 0.5 μM palbociclib for 48 h, 2.5 μM BYL719, 2.5 μM BKM120 or 50 nM BEZ235 for 24 h or were pre-incubated with palbocilcib for 24 h and then with palbociclib combined with the PI3K/mTOR inhibitors for further 24 h. Then the cells were stained with PI and the distribution of cells in cell cycle phases was determined by flow cytometry. Results are representative of three independent experiments (n = 5). *p < 0.05, **p < 0.01, ***p < 0.001 vs G0/G1 ctrl; ^**#**^p < 0.05 vs G0/G1 Palb; ^§§§^p < 0.001 vs G0/G1 BYL719, BEZ235 or BKM120. **h** The cells were treated as in **g** and the expression of the indicated proteins was evaluated on cell protein extracts by Western blotting. Results are representative of two independent experiments

**Fig. 6:**
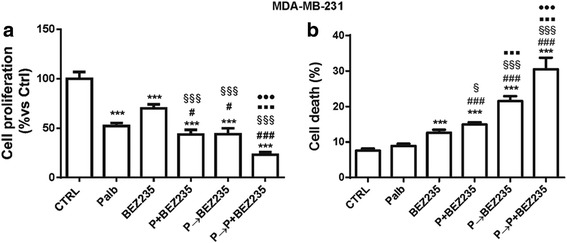
The combination of palbociclib with BEZ235 enhances cell death. MDA-MB-231 cells were treated with 0.5 μM palbociclib, 50 nM BEZ235 or with three schedules of treatments: simultaneous, sequential or sequential combined. After 72 h, cell proliferation was evaluated by cell counting with trypan blue exclusion (**a**) and cell death was assessed by fluorescence microscopy after Hoechst 3342/PI staining (**b**). Results are representative of three independent experiments (n = 5). ***p < 0.001 vs ctrl; ^#^p < 0.05, ^###^p < 0.001 vs palb; ^§^p < 0.05, ^§§§^p < 0.001 vs BEZ235; ^▪▪▪^p < 0.001 vs P + BEZ235; ^●●●^p < 0.001 vs P➔BEZ235

These findings prompted us to investigate the efficacy of combining palbociclib with three PI3K/mTOR inhibitors: BYL719, an inhibitor of the p110α catalytic subunit of PI3K, BEZ235, a dual PI3K and mTORC1–2 inhibitor, and BKM120, a pan-class I PI3K inhibitor. The exposure of TNBC cell lines to these compounds resulted in the inhibition of cell proliferation as shown in Additional file 1: Table S1. We initially evaluated the simultaneous combination of increasing concentrations of the PI3K/mTOR inhibitors with a fixed concentration of palbociclib (0.5 μM) on MDA-MB-231 (Fig. 4a-c) and HCC-38 cells (Fig. 4d-f). Such combination gave rise to an additive effect according to Bliss experimental model. Since AKT activation increased up to 24 h of treatment with palbociclib, we then evaluated the efficacy of a combinatory schedule involving a pre-incubation with palbociclib for 24 h followed by a simultaneous treatment with the PI3K inhibitors plus palbociclib for further 48 h (sequential combined treatment). As shown in Fig. 5a-f, this sequential combined approach resulted in a synergistic inhibition of cell proliferation in both MDA-MB-231 and HCC-38 cells. Of note, when palbociclib was removed during treatment with PI3K inhibitors the synergism was lost, resulting in an additive inhibition of cell proliferation (Additional file 2: Figure S1). The sequential combined treatment was associated with a greater increase of the percentage of cells accumulating in the G0/G1 cell cycle phase (Fig. 5g), with values of ~ 90% for all the PI3K inhibitors used. In addition, the sequential combined treatment induced a stronger down-regulation of the PI3K/AKT/mTOR pathway, as shown by the inactivation of mTOR (Fig. 5h), together with a complete dephosphoryation of p-Rb, in comparison with the single drug treatments. Moreover, in these conditions myc expression was strongly inhibited due to Rb inactivation. Directly comparing the growth-inhibitory effects induced by the three different schedules of treatment, we confirmed the superior efficacy of the sequential combined treatment over the others (23% of cell proliferation vs 43–44% for the combined and the sequential treatments); in addition, palbociclib alone did not induce cell death, as expected, mainly exerting a cytostatic effect; however, its combination with BEZ235 potentiate the pro-apoptotic effects associated with PI3K/AKT/mTOR inhibition, with the sequential combined treatment showing the stronger cell death induction (31% of cell death vs 15% and 21% for the combined and the sequential treatment, respectively) (Fig. 6a,b).

Taken together these results indicate that palbociclib may be used to potentiate the anti-tumor activity of PI3K inhibitors, and suggest the relevance of maintaining palbociclib during treatment with these inhibitors to provide the greatest benefit for TNBC.

### Effect of palbociclib and PI3K/mTOR inhibition on glucose energy metabolism

**Fig. 7:**
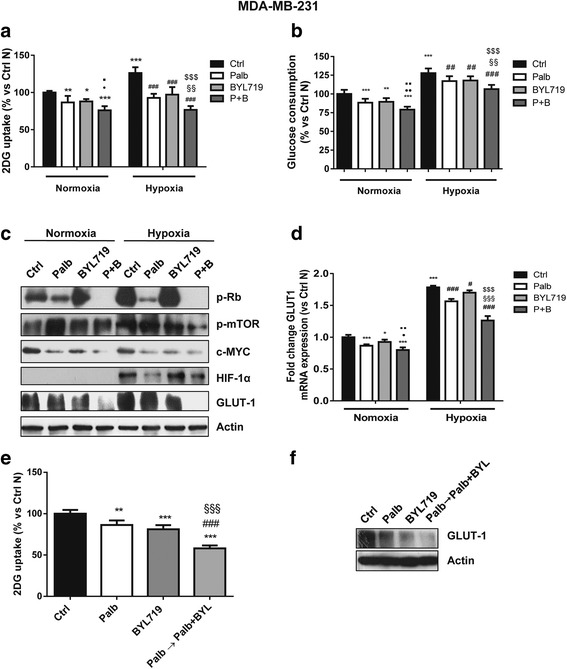
Palbociclib combined with the PI3K/AKT inhibitor BYL719 hinders glucose metabolism under normoxic and hypoxic conditions. MDA-MB-231 cells were treated with 0.5 μM palbociclib and 5 μM BYL719 alone or in combination under normoxic or hypoxic (1% O_2_) conditions for 24 h. Glucose uptake (**a**) and glucose consumption (**b**) were measured. *p < 0.05, **p < 0.01, ***p < 0.001 vs ctrl Normoxia (N); ^●^p < 0.05, ^●●^p < 0.01 vs palb N; ^▪^p < 0.05, ^▪▪^p < 0.01 vs BYL N; ^##^p < 0.01, ^###^p < 0.001 vs ctrl Hypoxia (H); ^§§^p < 0.01 vs Palb H; ^$$$^p < 0.001 vs BYL H. **c** The expression of the indicated proteins was analyzed by Western blotting. **d** GLUT-1 mRNA levels were analyzed by RT-PCR. Results are plotted as 2^-ΔΔCT ± SD. **e** MDA-MB-231 cells were treated with 0.5 μM palbociclib for 48 h and 5 μM BYL719 for 24 h alone or were pre-incubated with palbocilcib for 24 h and then with palbociclib combined with BYL719 for further 24 h. Glucose uptake was then evaluated. **p < 0.01, ***p < 0.001 vs ctrl; ^###^p < 0.001 vs palb; ^§§§^p < 0.001 vs BYL. **f** The cells were treated as in **e** and GLUT-1 expression was evaluated by Western blotting on cell protein extracts. Data in **a**,**b** and **e** are mean values ±SD of three independent experiments (*n* = 6). Data in **d** are mean values ±SD of three independent experiments (*n* = 4). Results in **c** and **f** are representative of two independent experiments

A variety of evidence indicates that cell cycle-related proteins are directly involved in the regulation of cell energy metabolism [24]. Therefore, we evaluated the effect of palbociclib, alone or combined with BYL719, on cell glucose metabolism under both normoxic and hypoxic conditions (Fig. 7a-d). Palbociclib down-regulated glucose uptake and consumption, as well as the expression of GLUT-1 glucose transporter in MDA-MB-231 cells, and combination with BYL719 enhanced these inhibitory effects under normoxic conditions. In particular, palbociclib induced ~ 15% decrease in glucose uptake and consumption, the same produced by the treatment with BYL719 alone; a higher decrease (25%) was promoted by the drug combination. Exposure to hypoxia for 24 h induced the stabilization and accumulation of HIF-1α and up-regulated the expression of GLUT-1, resulting in a significant increase of glucose utilization. Again, the combination of palbociclib with BYL719 hindered hypoxia-mediated stimulation of glucose uptake more efficaciously than individual treatments (40% decrease vs 25% after palbociclib or BYL719 single treatment). Similar effects were observed for glucose consumption. The greater efficacy of such combination was presumably ascribed to the inhibition of both PI3K/mTOR signaling and c-myc expression (see Fig. 5h), whose involvement in the modulation of glucose metabolism is well-recognized [25]. Interestingly, the sequential combined treatment with palbociclib maintained during the incubation with BYL719 promoted a ~ 50% decrease of glucose uptake as compared to control (Fig. 7e), which was associated with a significant inhibition of GLUT-1 expression (Fig. 7f).

Altogether these results suggest that inhibition of PI3K/mTOR signaling may improve the efficacy of palbociclib also through a negative modulation of glucose metabolism.

## Discussion

In the present study we demonstrate that CDK4/6 inhibition by palbociclib in combination with PI3K/mTOR inhibitors may be an effective strategy for treatment of Rb-proficient TNBC, affecting both cell proliferation/viability and energy metabolism.

A functional Rb pathway is more frequently found in luminal A hormone-positive BC, and preclinical data have indicated these tumors as exquisitely sensitive to CDK4/6 inhibition, providing the basis for the development of novel therapeutic strategies for this BC subtype [22]. Actually, palbociclib has received accelerated approval for first-line treatment in combination with letrozole or fulvestrant in postmenopausal women with ERα+/HER2 − locally advanced or metastatic BC. In contrast, TNBC has been considered less likely to respond to CDK4/6 inhibitors [22], being frequently associated with Rb loss [26]. However, a number of preclinical data have been suggesting that palbociclib treatment may provide benefit also in a proportion of TNBC [12, 27]. Here we show that palbociclib as a single-agent treatment reduced cell proliferation in TNBC cell models, by eliciting reversible blockade of the cell cycle in G1 phase. This effect was observed only in cell lines expressing the predictive markers of response to palbociclib, i.e. Rb, cyclin D1, and CDK6 proteins, associated with undetectable levels of p16^INK4^. Palbociclib treatment in these cells inhibited p-Rb, Rb, and p-CDK6 levels, and more interestingly down-regulated the expression of c-myc, a direct target of E2F transcription factor. Very recently, CDK4 has been found highly expressed in TNBC, especially in the basal-like subtype [27], and this feature may serve as an additional predictive marker of response to palbociclib in this type of BC. Blocking CDK4 expression or activity in TNBC cells has been shown to prevent BC stem cell self-renewal [27], thus providing a further rationale for exploiting palbociclib as a therapy for TNBC. In addition, it has been recently demonstrated, in BC cell lines including TNBC cells, that treatment with CDK4/6 inhibitors triggers anti-tumor immunity by enhancing tumor antigen presentation and suppressing regulatory T cells proliferation [28], thus opening new perspectives in the use of CDK4/6 inhibitors in association with immunotherapies.

In our study, palbociclib treatment gave the most promising results when combined with PI3K/mTOR inhibitors.

A relevant feature of palbociclib treatment is the induction of AKT activation, through the release of p-Rb-mediated suppression of mTORC2. Hyperphosphorylated Rb inhibits the activity of the mTORC2 complex, by directly binding to the complex component Sin1. Since AKT is a substrate of mTORC2, the inhibition of Rb phosphorylation, associated with CDK4/6 inhibition, results in mTORC2 activation, thus increasing AKT activation [13].

This provided a rationale for evaluating the efficacy of combining palbociclib with PI3K/mTOR inhibitors following different schedules of treatment: a simultaneous combination, a sequential treatment in which a pre-incubation with palbociclib was followed by treatment with PI3K/mTOR inhibitors alone, or a sequential combined treatment in which palbociclib, given as a pre-treatment, was also maintained during exposure to PI3K/mTOR inhibitors. The latter schedule produced a synergistic growth inhibitory effect as calculated by the Bliss experimental model, whereas an additive effect was observed with the other two schedules.

The synergistic effect of the association of palbociclib and PI3K inhibitors has been recently reported for luminal androgen receptor-positive TNBC cell lines and has been associated with low level of CDK2 activity, rather than with p16^INK4^ loss and cyclin D1 expression [12]. Here we show that also other TNBC subtypes, i.e. mesenchymal and basal-like subgroups, represented by MDA-MB-231 and HCC38 cell models respectively [29], may benefit from a combined therapy with palbociclib and PI3K/mTOR inhibitors, provided that they express the predictive factors of response to palbociclib. Importantly, our results demonstrate that the sequential combined treatment of palbociclib and PI3K/mTOR inhibitors is more effective than simultaneous treatment and might therefore represent a novel therapeutic approach for the treatment of TNBC of different cell subtypes.

Aberrant energy metabolism is an important hallmark of cancer, that is being exploited as a therapeutic target [30]. Here we provide evidence that inhibition of glucose metabolism may contribute to palbociclib anti-tumor activity in TNBC cells. The cyclin D1/CDK6/Rb/E2F pathway has been involved in the control of a variety of metabolic processes, such as glucose production and glycolytic metabolism, indicating a close relationship between metabolic responses and proliferative stimuli [31]. There is evidence that E2F pathway is a negative regulator of energy expenditure, through repression of mitochondrial oxidative metabolism [24]; E2F is also able to stimulate the glycolytic flux through regulation of phosphofructokinase enzyme expression [24]. In addition, downstream of E2F, c-myc transcription factor may affect cancer metabolic reprogramming through a variety of mechanisms, and in particular it has been shown to drive glucose metabolism in TNBC cells [32].

Here we demonstrate that palbociclib-mediated inhibition of E2F/c-myc hinders glucose metabolism in TNBC cells, by inhibiting GLUT-1 expression and glucose uptake under both normoxic and hypoxic conditions. c-Myccooperates with HIF-1 to induce the expression of glycolytic enzymes [33]; interestingly, palbociclib inhibited also hypoxia-induced HIF-1α accumulation, which might contribute to reduce glucose utilization under hypoxia. This result is in line with a recent study showing that palbociclib destabilizes HIF-1α in colon cancer cells under either normoxic or hypoxic conditions [34]. In our study, the inhibitory effects of palbociclib on glucose metabolism were further enhanced by the combination with PI3K/mTOR inhibitors, being the AKT/mTOR signaling another key player in cancer metabolic reprogramming [35]. Accordingly, palbociclib combined with the mTOR inhibitor everolimus has been recently demonstrated to inhibit aerobic glycolysis in glioblastoma cells [36]. Importantly, we observed a strong impairment of glucose metabolism when the combination with palbociclib and PI3K/mTOR inhibitors was preceded by treatment with palbociclib alone, likely contributing to the synergistic anti-tumor effects associated with this schedule of treatment.

## Conclusions

In conclusion, our study provides a pre-clinical rationale for the combination of palbociclib with PI3K/mTOR inhibitors as a therapeutic strategy for TNBC, highlighting the superior efficacy of the sequential combined schedule of treatment. A limitation of this study is that these results were obtained in in vitro experiments and would require confirmation in vivo in animal models, also to ascertain whether palbocilcib may be safely maintained during treatment with PI3K/mTOR inhibitors in order to produce synergistic anti-tumor effects without worsening toxicity.

## Additional files

Additional file 1: **Table S1.** Sensitivity of TNBC cells to PI3K/mTOR inhibitors. HCC38 cells and MDA-MB-231 cells were treated with increasing concentrations of the indicated PI3K/mTOR inhibitors. After 72h cell proliferation was evaluated by CV staining. Data are expressed as EC_50_ values and are means ±SD of three independent expreiments (*n* = 5). (TIFF 371 kb)

Additional file 2: **Figure S1.** A sequential exposure to palbociclib followed by PI3K/AKT/mTOR inhibitors alone induces additive effects. MDA-MB-231 were pre-incubated with 0.5 μM palbociclib for 24h and then treated with increasing concentrations of BYL719 (**a**), BEZ235 (**b**) or BKM120 (**c**) alone. After 48h cell proliferation was assessed by CV assay. The effect of the drug combinations was evaluated using the Bliss interaction model. Data are mean values ±SD of three insdependent experiments (*n* = 5). (TIFF 437 kb)

## Abbreviations

CDK: Cyclin-dependent kinases
RB: Retinoblastoma Protein
TNBC: Triple-negative Breast Cancer
