# Correction: The anti-tumor efficacy of CDK4/6 Inhibition is enhanced by the combination with PI3K/AKT/mTOR inhibitors through impairment of glucose metabolism in TNBC cells

**DOI:** 10.1186/s13046-025-03383-x

**Published:** 2025-04-16

**Authors:** Daniele Cretella, Andrea Ravelli, Claudia Fumarola, Silvia La Monica, Graziana Digiacomo, Andrea Cavazzoni, Roberta Alfieri, Alessandra Biondi, Daniele Generali, Mara Bonelli, Pier Giorgio Petronini

**Affiliations:** 1 Department of Medicine and Surgery, University of Parma, Parma, Italy; 2 Department of Medical, Surgery and Health Sciences, University of Trieste, Trieste, Italy; 3 U.O. Multidisciplinare di Patologia Mammaria, U.S Terapia Molecolare e Farmacogenomica, ASST Cremona, Cremona, Italy

**Correction: J Exp Clin Cancer Res 37**,** 72 (2018)**

10.1186/s13046-018-0741-3

Following the publication of the original article [1], the authors identified errors in Figs. 2 and 3. Blots were developed using the method with films and these errors could possibly due to incorrect film was scanned twice and/or in the wrong side and was mislabeled.

The correct figures are presented below:

**Incorrect Figure**  2

**Fig. 2:**
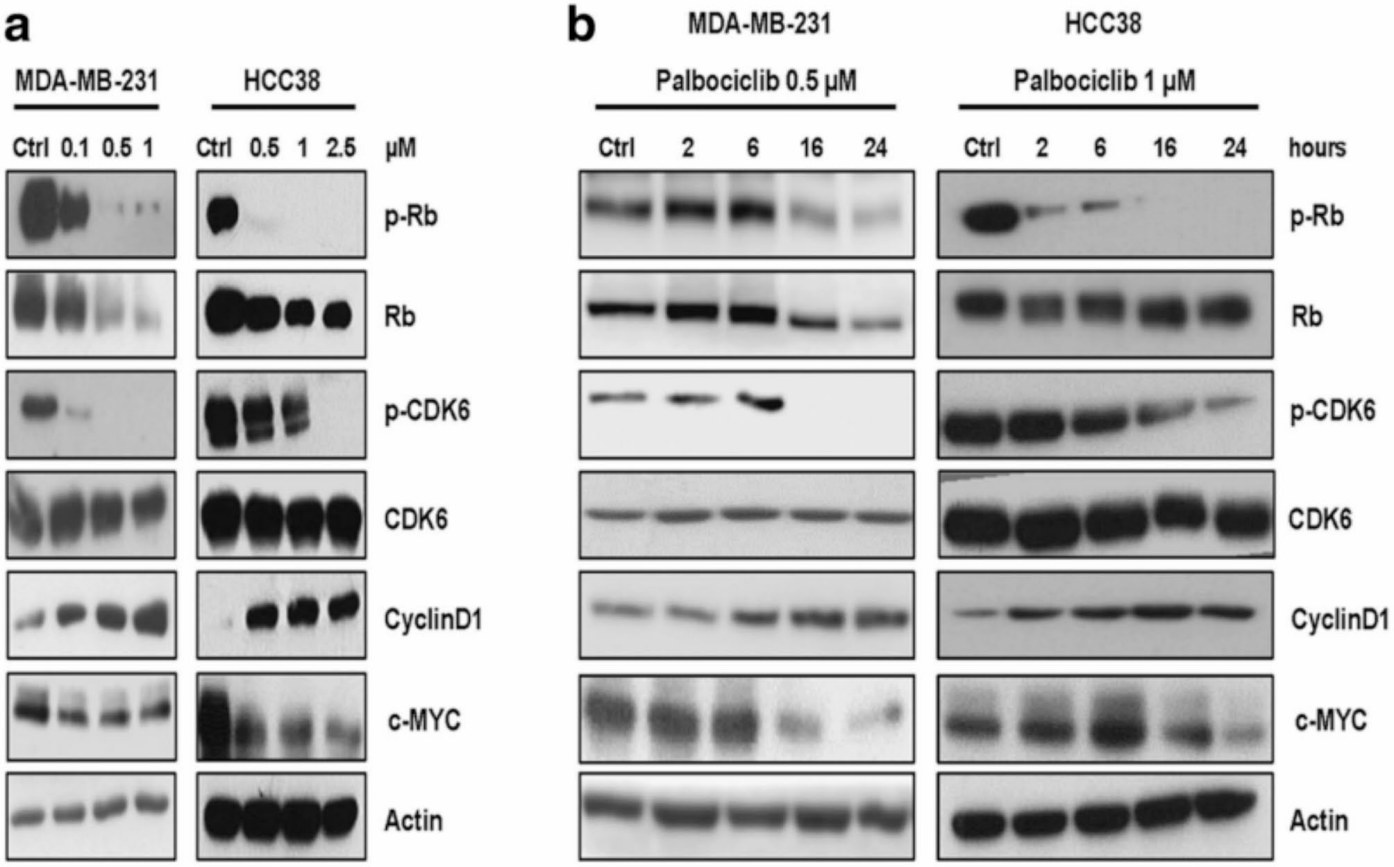
Palbociclib modulates the activation/expression of cell cycle-related proteins in a dose- and time-dependent manner. MDA-MB-231 and HCC38 cells were treated with increasing concentrations of palbociclib for 24 h (**a**) or with a fixed drug concentration for different periods of time (**b**). The expression of the indicated proteins was analyzed by Western blotting. Results are representative of three independent experiments

**Correct Figure**  2

**Fig. 2:**
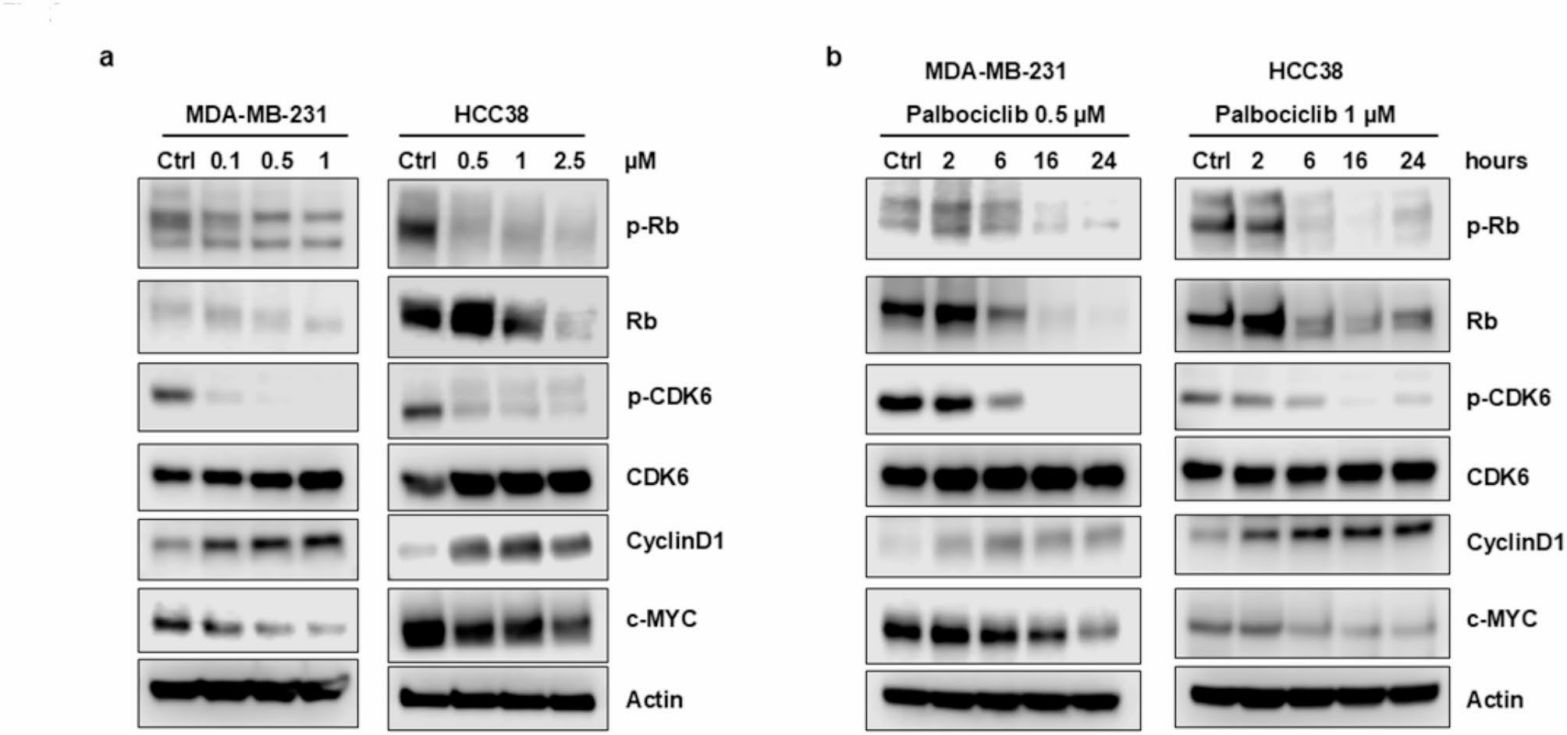
Palbociclib modulates the activation/expression of cell cycle-related proteins in a dose- and time-dependent manner. MDA-MB-231 and HCC38 cells were treated with increasing concentrations of palbociclib for 24 h (**a**) or with a fixed drug concentration for different periods of time (**b**). The expression of the indicated proteins was analyzed by Western blotting. Results are representative of three independent experiments

**Incorrect Figure**  3

**Fig. 3:**
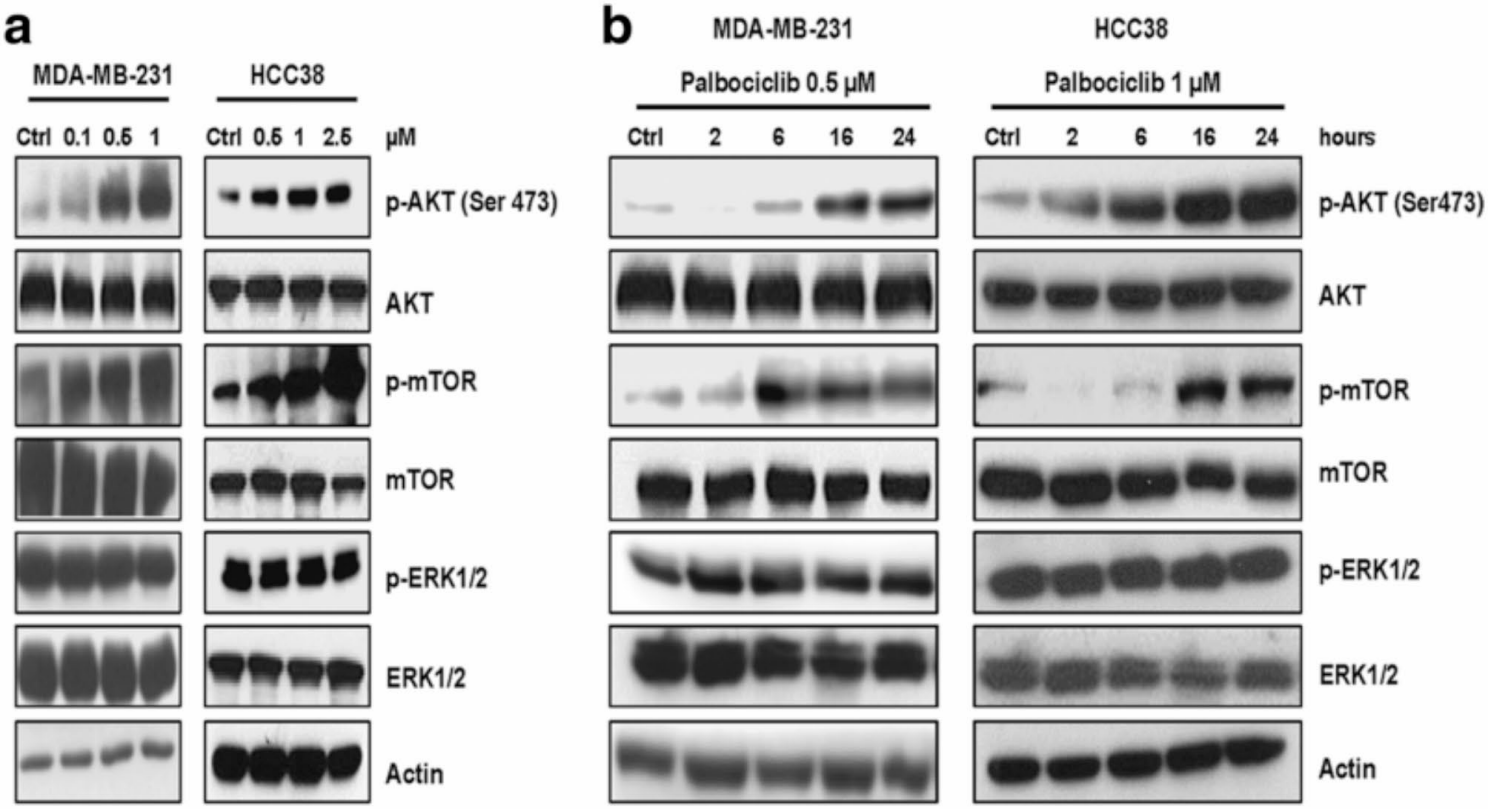
Palbociclib up-regulates the PI3K/AKT/mTOR pathway in TNBC cells. MDA-MB-231 and HCC38 cells were treated with increasing concentrations of palbociclib for 24 h (**a**) or with a fixed drug concentration for different periods of time (**b**). Then, the expression of the indicated proteins was analyzed by Western blotting. Results are representative of three independent experiments

**Correct Figure**  3

**Fig. 3:**
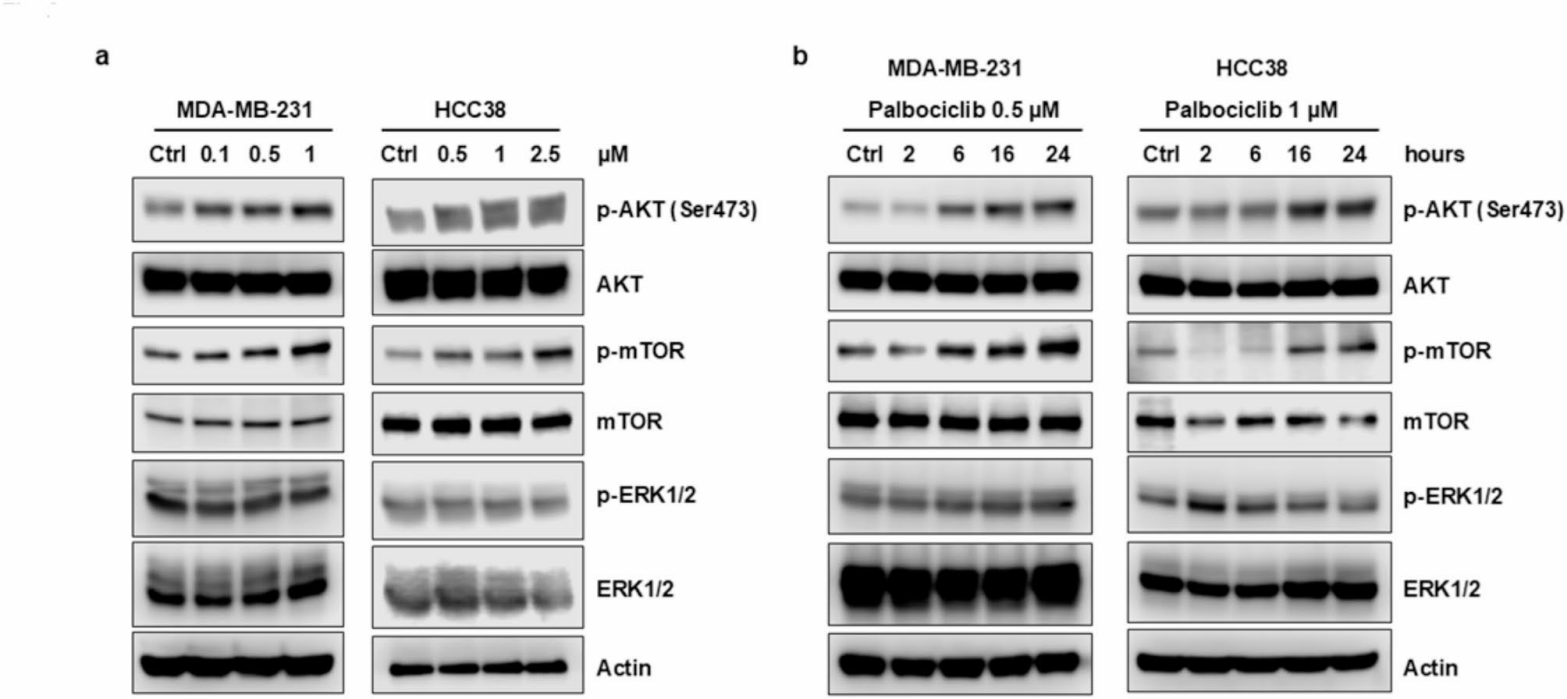
Palbociclib up-regulates the PI3K/AKT/mTOR pathway in TNBC cells. MDA-MB-231 and HCC38 cells were treated with increasing concentrations of palbociclib for 24 h (**a**) or with a fixed drug concentration for different periods of time (**b**). Then, the expression of the indicated proteins was analyzed by Western blotting. Results are representative of three independent experiments

The corrections do not compromise the validity of the conclusions and the overall content of the article.
