# Supplementary figures and images for: The anti-tumor efficacy of CDK4/6 inhibition is enhanced by the combination with PI3K/AKT/mTOR inhibitors through impairment of glucose metabolism in TNBC cells
Source: J Exp Clin Cancer Res. 2018 Mar 27;37:72. doi: 10.1186/s13046-018-0741-3 (PMC5872523; doi:10.1186/s13046-018-0741-3)

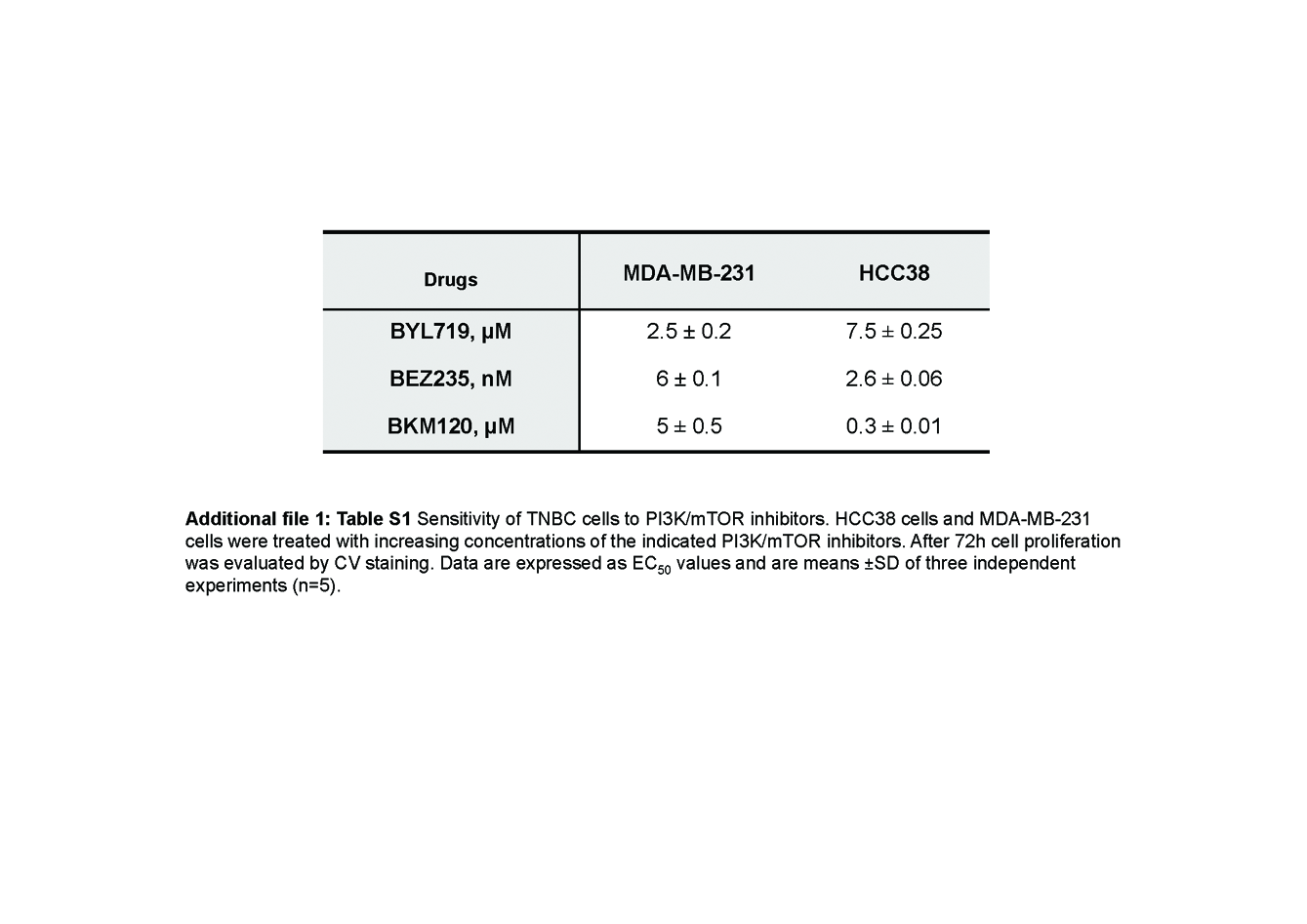

Supplement: Supplementary file 1 — Table S1. Sensitivity of TNBC cells to PI3K/mTOR inhibitors. HCC38 cells and MDA-MB-231 cells were treated with increasing concentrations of the indicated PI3K/mTOR inhibitors. After 72h cell proliferation was evaluated by CV staining. Data are expressed as EC50 values and are means ±SD of three independent expreiments (n = 5). (TIFF 371 kb) [file 13046_2018_741_MOESM1_ESM.tif]

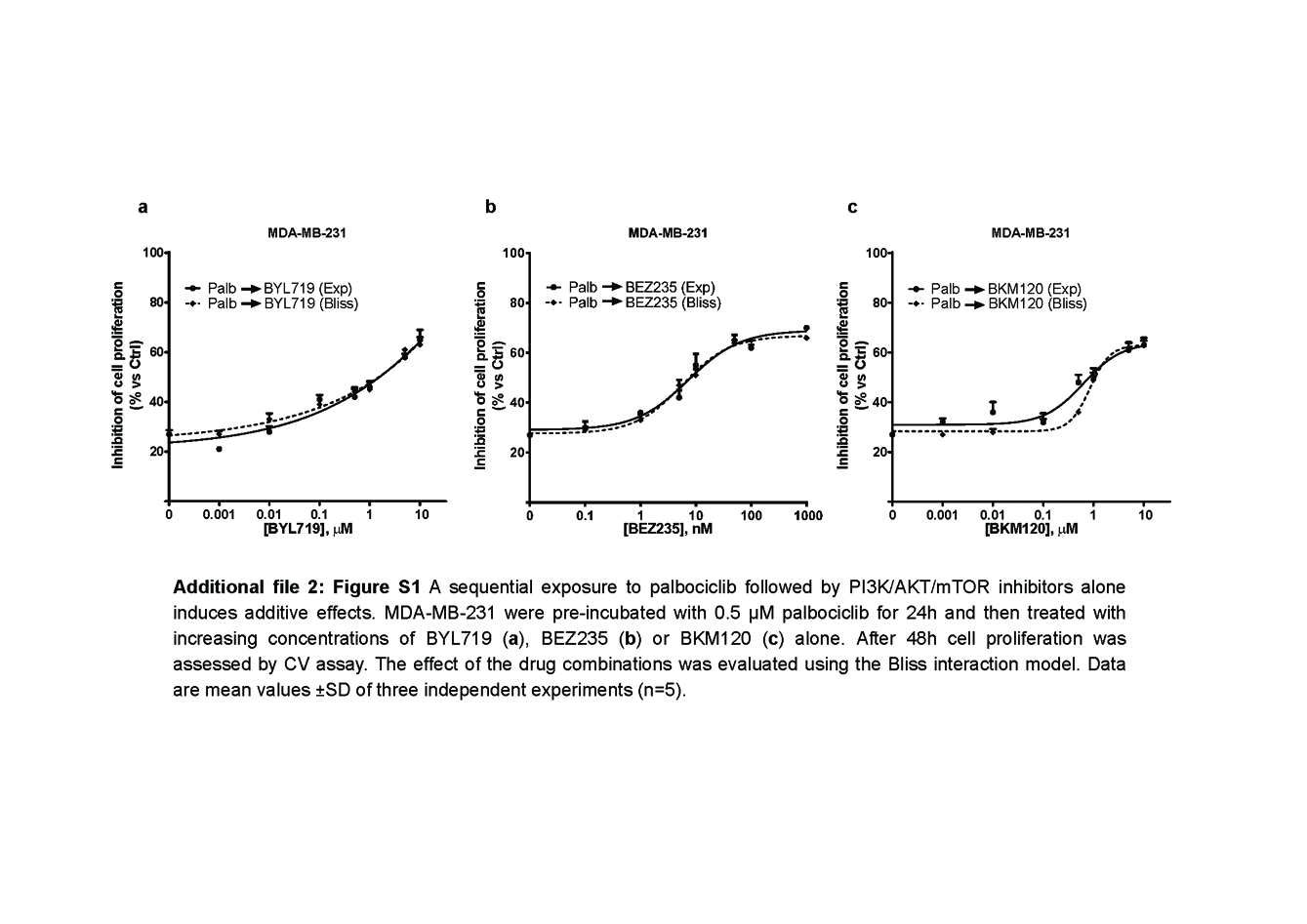

Supplement: Supplementary file 2 — Figure S1. A sequential exposure to palbociclib followed by PI3K/AKT/mTOR inhibitors alone induces additive effects. MDA-MB-231 were pre-incubated with 0.5 μM palbociclib for 24h and then treated with increasing concentrations of BYL719 (a), BEZ235 (b) or BKM120 (c) alone. After 48h cell proliferation was assessed by CV assay. The effect of the drug combinations was evaluated using the Bliss interaction model. Data are mean values ±SD of three insdependent experiments (n = 5). (TIFF 437 kb) [file 13046_2018_741_MOESM2_ESM.tif]
